# Testosterone Replacement Therapy Is Not Associated with Greater Revision Rates in Reverse Total Shoulder Arthroplasty

**DOI:** 10.3390/jcm14041341

**Published:** 2025-02-18

**Authors:** Romir P. Parmar, Austin Cronen, Clayton Hui, Michael Stickels, Evan Lederman, Anup Shah

**Affiliations:** 1 Department of Orthopaedic Surgery, The University of Arizona College of Medicine-Phoenix, 475 N 5th St., Phoenix, AZ 85004, USA; amcronen@arizona.edu (A.C.); claytonhui@arizona.edu (C.H.); mstix98@arizona.edu (M.S.); 2 Department of Orthopaedic Surgery, Banner University Medical Center-Phoenix, 1111 E McDowell Rd, Phoenix, AZ 85006, USA; evan.lederman@bannerhealth.com (E.L.); anup.shah@bannerhealth.com (A.S.)

**Keywords:** testosterone, hormone therapy, shoulder arthroplasty, gender-affirming therapy, osteoarthritis, rotator cuff arthropathy, surgical complications

## Abstract

**Background/Objectives**: Testosterone replacement therapy (TRT) has become increasingly common, particularly for patients with symptomatic hypogonadism or individuals undergoing gender-affirming therapy. The current literature is inconclusive on the association between TRT and orthopedic surgery. This study sought to examine outcomes of reverse total shoulder arthroplasty (RSA) in patients receiving TRT. **Methods**: A retrospective cohort of RSA patients from 2010 to 2022 was queried using the PearlDiver database. Patients were included if they underwent RSA with at least 2 years of follow-up. Patients who underwent at least 90 days of TRT prior to their surgery were matched by Charlson Comorbidity Index, age, and gender to a control cohort. Univariate analysis using chi-squared tests and Student’s *t*-tests were used to compare demographics outcomes between groups. **Results:** A total of 1906 patients were identified who used TRT within 90 days of undergoing RSA, and these patients were matched to a control cohort of 1906 patients. Patients who used TRT within 90 days did not have significantly different rates of revision RSA (12.01%) compared to those without use (11.02%) (*p* = 0.335). Furthermore, between the TRT group and the control group, PJI rates (1.42% vs. 1.63%; *p* = 0.597) and periprosthetic fracture rates (0.58% vs. 1.05%, *p* = 0.105) were not significantly different. **Conclusions**: This study demonstrated that TRT use within 90 days of RSA does not increase the rates of revision, fracture, or infection. These results can assist surgeons when evaluating patients on TRT who also may be candidates for RSA.

## 1. Introduction

Reverse shoulder arthroplasty (RSA) is a key treatment for conditions such as rotator cuff arthropathy, proximal humeral fractures, and advanced glenohumeral osteoarthritis, even in the presence of an intact rotator cuff, particularly when associated with glenoid retroversion, posterior humeral subluxation, and glenoid bone loss [1,2,3]. In recent years, the volume of RSAs performed has exponentially risen, with projections to continue over the following years [4]. While historically performed in older populations, the use of RSA in patients younger than 65 is increasing and has been shown to remain safe and effective in appropriately selected cases, contributing to the sharp rise in the number of surgeries performed [5]. As RSA approaches its 20-year anniversary in the United States, implant survivorship has been increasingly studied, with a focus on implant design and placement and patient selection. As cuff tear arthropathy continues to remain a common etiology of shoulder pain in the general population [6], and in those above the age of 60, continuing to evaluate preoperative patient variables that can affect RSA outcomes has become increasingly essential [7].

Testosterone replacement therapy (TRT) has become more prevalent in recent decades for both hypogonadism and gender-affirming care [8,9]. With an increasing population over 65 years of age, the number of men who experience age-related androgen decline will also increase [10]. Hypogonadism in males is a clinical syndrome resulting in decreased muscle mass, bone mass, libido, and sexual desire, and TRT is the standard prescription for these symptoms [11]. Although TRT is most common in males older than 30 years of age, a recent study demonstrated a three-fold increase in TRT use in men aged 56–64 from 2003 to 2013 [12]. Given overlapping age and other demographics, the volume of patients undergoing RSA may also be on TRT. TRT is also used for female-to-male gender transitions, where it has proven to be very successful in providing masculinizing effects [13,14]. The number of transgender patients in the United States is estimated to be around 1.5 million, with the numbers expected to increase over the next decade [15]. Given the prevalence of transgender individuals who may be on TRT during the perioperative period of an orthopedic procedure, understanding the proper management of these patients is important. With respect to arthroplasties, there remains a gap in knowledge of the risks that hormonal therapies may have on the success of a surgery like RSA [16]. While there is not much research out on RSA, a retrospective database study investigating the effects of hormone replacement therapy on outcomes of total hip arthroplasty and total knee arthroplasty found that patients who received TRT demonstrated significantly higher rates of revision and prosthetic joint infection [17]. As more individuals begin to age into joint replacements, it is probable that orthopedic surgeons will treat these patients, underscoring the need to elucidate the effects of the long-term use of TRT [18].

TRT’s impact on the musculoskeletal system is well known, as increased trabecular bone mineral density and strength is associated with testosterone use [19,20]. Contrarily, TRT can also have negative impacts on soft tissue, such as tendons, due to impaired regulation of matrix metallopeptidase [21]. While there is established research on the basic science of the musculoskeletal system, there remains a paucity of literature on the effect of TRT on the clinical outcomes of orthopedic shoulder surgeries [22]. One study demonstrated an increased risk of prosthetic joint infection following any form of total shoulder arthroplasty (TSA) in patients who were on TRT [23]. Another study found a higher risk of rotator cuff pathology in patients who were prescribed testosterone [24]. To our knowledge, there are no current studies that investigate the effect of TRT on patients undergoing RSA. The primary outcome of this study was to determine the rates of revision RSA following surgery in patients who used TRT within 90 days of initial surgery. Secondary outcomes include evaluations of specific surgical complications requiring revision, like prosthetic joint infection (PJI), broken prosthesis, periprosthetic fracture, and mechanical loosening.

## 2. Materials and Methods

A retrospective cohort study was conducted using the PearlDiver Mariner database (PearlDiver Technologies, Colorado Springs, CO, USA) from 2010 to 2022. The PearlDiver database contains Humana Insurance claims data for more than 150 million patients and tracks the patients over time using diagnosis and procedure. This study queried the database in order to identify the patient cohorts, including patients undergoing RSA, and those that received TRT. Since PearlDiver is a deidentified database, this study was exempt from institutional review board approval.

The database was queried for all patients who underwent RSA with at least two years of follow-up. It was also queried for all individuals who were taking a form of TRT, which was defined by getting a prescription of any form of testosterone. The specific International Classification of Diseases (ICD) codes and Current Procedural Terminology (CPT) codes are defined in Supplementary Table S1. Of note, CPT codes were unable to be used to identify patients who underwent RSA because the current CPT codes used in shoulder arthroplasty include all forms of shoulder arthroplasty. The following exclusion criteria for TRT were used: autoimmune disease, connective tissue disorders, and mitochondrial diseases (Supplementary Table S2) [24]. Individuals with these disorders were excluded from this study, as TRT could exacerbate or complicate these conditions by altering immune function, inflammation, collagen metabolism, metabolic processes, and the musculoskeletal system, thereby acting as a confounder to the outcomes. To ensure there was proper follow-up of patients, patients who were on TRT must have had active medical records 1 year before and 2 years after the prescription was filled. Patients over the age of 18 of either biological sex were included from the years 2010 to 2022. A total of 98,782 patients who underwent RSA with at least 2 years of follow-up were identified. A total of 1,804,865 patients who were on TRT in the same time period were identified. For the present study, patients who were on TRT within 90 days of undergoing RSA were included (1906 patients). A matched population of those not on TRT but who still underwent RSA was created with propensity score matching using age, Charlson Comorbidity Index, and gender as the variables. The Charlson Comorbidity Index is an index that is widely used throughout medicine that helps predict 10-year survival in patients with many comorbidities [25]. It has been used in the orthopedic surgery literature as well [26,27]. Patient demographics, comorbidities, and surgical complications were compared between the RSA group and the control group to determine if TRT use within 90 days of surgery has any effect on the surgical outcomes.

Patient comorbidities were identified for both the TRT group and control group. The comorbidity must have been present prior to and after the RSA. The comorbidities were either identified by using pre-defined groups of codes within PearlDiver, or by codes that the authors identified (Supplementary Table S3). Diabetes, tobacco use, osteoarthritis, chronic kidney disease, alcohol use, obesity, congestive heart failure, and dementia all had pre-defined codes. The codes for hypogonadism, erectile dysfunction, decreased libido, and benign prostatic hyperplasia were identified by the authors.

Surgical complications were identified for both groups as well. These included a revision shoulder arthroplasty procedure, prosthetic joint infection, broken prosthesis, periprosthetic fracture, and mechanical loosening. These codes are shown in Table S4. It was ensured that these complications occurred after the index surgery in order to prevent confounding factors or diagnoses that may have been made prior to the surgery.

Descriptive statistics were reported where appropriate, including means, frequencies, and proportions. In order to compare the demographics and surgical complications of the TRT and control groups, chi-squared and Student’s t-tests were used for a univariate analysis. Standard errors were reported, where appropriate. Statistically significant differences were noted if *p* < 0.05. All analyses were conducted using R, version 4.2.3 (R Core Team, Vienna, Austria).

## 3. Results

### 3.1. Demographics and Patient Comorbidities

There were a total of 1906 patients who used TRT within 90 days of undergoing RSA and at least 2 years of follow-up after surgery. The demographics and comorbidities of the TRT use and control cohort are shown in Table 1. Both groups contained a majority of biological males (89.5%), with an average age of 68 years. Furthermore, patients who were using TRT did not have a statistically different Charlson Comorbidity Index score compared to those not using TRT (2.78 ± 2.35 vs. 2.78 ± 2.33). Patients on TRT were significantly more likely to have a concurrent diagnosis of hypogonadism (67.3% vs. 9.3%; *p* < 0.001), decreased libido (11.2% vs. 3.8%; *p* < 0.001), erectile dysfunction (35.5% vs. 18.3%, *p* < 0.001), and benign prostatic hyperplasia (33.8% vs. 25.8%; *p* < 0.001) than patients in the control group. There was also a higher prevalence of obesity (46.2% vs. 39.5%; *p* < 0.001), diabetes (51.8% vs. 48.3%; *p* < 0.03), chronic kidney disease (22.0% vs. 18.4%; *p* < 0.006), and dementia (2.8% vs. 1.8%; *p* < 0.04) in the TRT group as compared to the control group. Between the groups, no statistical difference was observed when comparing tobacco use, alcohol abuse, congestive heart failure, and osteoarthritis (*p* > 0.05).

### 3.2. Effect of TRT Use on Outcomes of RSA

**Figure 1:**
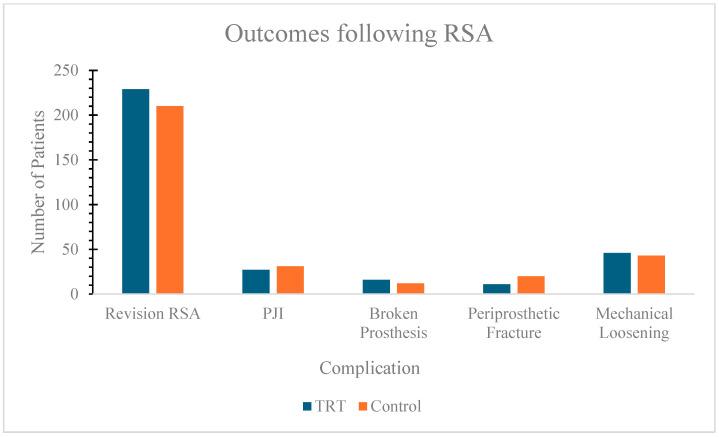
Graph demonstrating the complications in TRT group (n = 1906) and control group (n = 1906).

Outcomes for the TRT and control groups are shown in Table 2 and Figure 1. Patients who used TRT within 90 days of surgery did not have significantly different rates of revision RSA (12.0%) compared to those without use (11.0%) (*p* = 0.335). Between the TRT group and the control group, patients did not have a significantly increased risk of developing PJI (1.4% vs. 1.6%; *p* = 0.597) or an implant fracture (0.84% vs. 0.63; *p* < 0.448). Furthermore, there were no significant differences noted between groups when comparing periprosthetic fractures (0.58% vs. 1.05%; *p* = 0.105) and mechanical loosening (2.5% vs. 2.3%; *p* = 0.596).

## 4. Discussion

In the present study, a total of 1906 patients who used TRT within 90 days of RSA were identified from a national insurance claims database. Compared to a matched control cohort, patients who used TRT within 90 days did not have significantly different rates of revision RSA. Furthermore, between the TRT group and the control group, the rates of complications, like PJI, broken prosthesis, periprosthetic fracture, and mechanical loosening, were not significantly different. While several studies have reported on TSA complications with TRT, including prosthetic joint infection, this is the first large retrospective study to specifically assess the complications following RSA in patients taking testosterone.

Testosterone use has increased in recent years, particularly due to its beneficial impacts on sexual function [28], mood [29], muscle mass [30,31], and cognitive function [32] in patients with hypogonadism. While it can provide these benefits, its safety profile must also be addressed. Several clinical studies have investigated the potential risks in association with the cardiovascular [33], respiratory [34], urologic [35], reproductive [36], and hematologic [37] systems [38]. In addition to TRT’s benefits in treating hypogonadism, it also plays a critical role in the female-to-male gender transition process, which has risen in prevalence over the past decades [39]. Particularly, transgender males on testosterone experienced increased growth of facial and body hair, muscle mass, and libido [40], providing a positive effect on individuals’ mood [41]. Despite these benefits, the risks of TRT previously mentioned remain when given to transgender males. Given the potential risks and benefits associated with TRT, providers are advised to take all factors of the individual into account when prescribing TRT, regardless of the indication for medication [42]. One of the factors that does not have a strong consensus is the effect of TRT on orthopedic procedures, despite the presence of relevant basic science research on the musculoskeletal system.

A recent review article by Cohn et al. in 2024 highlighted the importance of investigating TRT’s effect on orthopedic conditions, particularly during the perioperative period [22]. Bone mineral density is one aspect of orthopedic care that has previously shown to be influenced by TRT, as testosterone’s metabolites have a direct impact on osteoblast differentiation [19]. A clinical trial conducted by Snyder et al. (2017) demonstrated that testosterone treatment was associated with significantly greater bone mineral density and strength, particularly in trabecular bone [20]. While not statistically significant, in the present study, the TRT group had lower rates of periprosthetic fracture after surgery compared to the control group (0.58% vs. 1.05%, respectively). Furthermore, another study demonstrated that screws coated with testosterone had a greater contact surface between the bone and the implant, indicating there may be an underlying physiologic impact on bone strength [43]. On the other hand, it has been demonstrated that testosterone could impair tissue remodeling of tendons due to the downregulation of matrix metallopeptidase [21]. This has also been seen clinically, where patients on TRT have a higher likelihood of injuring their Achilles tendon [44], rotator cuff tendon [24], or their distal biceps tendon [45]. While the subscapularis is not universally repaired following RSA [46], the surgeons who do decide to repair it may need to pay closer attention to the negative impacts that TRT could have on tendon integrity.

Compared to anatomic TSA, RSA success is based on osseous implant integration and optimal deltoid and scapulothoracic function, and less on soft tissue tendon integrity [1,47]. The most common indications for a revision RSA include instability of prosthesis, infection, and mechanical loosening, which all predominantly relate to the implant. The results of the present study demonstrated that rates of revision were similar between the TRT group (12.0%) and control group (11.0%). While TRT’s effect in orthopedics generally revolves around its positive effect in increasing bone mineral density, this study’s findings did not reveal a decrease in revision rates of RSA. This could be due to a variety of reasons. Particularly, prior research surrounding hormone therapy has shown that bone mineral density may be increased only in patients who have baseline testosterone levels below the reference range [48]. However, not all TRT patients in this retrospective study may have met these criteria. Furthermore, the amount of time that one is taking TRT may also be a reason. In addition, prior studies demonstrate that longer-term TRT (up to one year) has the greatest increase in bone mineral density [49]. Our study only looked at patients who were on TRT within 90 days of surgery. These two aspects may help explain the study’s findings of similar revision rates, despite the overall positive effect of TRT on bone mineral density.

These results also held true when comparing other surgical complications, like broken prosthesis and mechanical loosening. There is minimal research out on testosterone use in TSA outcomes, but there are studies that look at testosterone use in other joint arthroplasties, which had mixed results. A recent study queried a large prospective study and found that for patients undergoing total hip arthroplasty or total knee arthroplasty, rates of revision within two years of surgery were statistically higher in the TRT group compared to the control group [17]. Contrarily, Amory et al. (2003) conducted a controlled trial, where the treatment group received a testosterone intramuscular injection four times over a 3-week period prior to total knee arthroplasty [50]. Compared to the control group, the patients in the testosterone group had associated improvements in functional recovery, as defined by standing, walking, and stair climbing. Therefore, the current literature on TRT in arthroplasty in inconclusive. Furthermore, patients who used TRT within 90 days of surgery had PJI rates that were similar to the control group following RSA in the present study. While the literature is very limited, a study by Su et al. (2023) found that testosterone use within 180 days of surgery may be associated with greater rates of PJI in all TSA patients [23]. The present study used a shorter time frame, which could explain the differences in results, as a longer time of exposure to testosterone may increase the incidence of PJI. These findings could be related to the bacterial load of *C. acnes* on the epidermis of patients, as testosterone has previously shown to increase this [51].

There are a few limitations of this study. Primarily, the use of a large retrospective database comes with its own drawbacks. Since this study relies on the database, which is dependent on ICD and CPT coding, any errors made by providers in inputting these codes cannot be addressed; future prospective studies could help mitigate this limitation by allowing for more accurate control of such variables. Furthermore, since the goal of this study was to only investigate the reverse form of TSA, the CPT code for TSA was not used. This required the use of only an ICD code that refers to the RSA procedure, which may have led to an under-reporting of the total number of RSA patients that may have also been on TRT. However, this allowed for the specification of the exact procedure of interest. Furthermore, when coding for periprosthetic fractures, fractures due to falls after surgery compared to intraoperative fractures was not able to be elucidated. While bone–implant integration is a critical aspect of RSA success, this study was unable to account for uncemented vs. cemented implants. Furthermore, this study is also limited by the lack of documentation regarding the type of prosthesis used, the use of grafts, the stage of arthropathy, and glenoid classification, as well as the absence of clinical outcomes such as patient-reported outcome measures, range of motion, and radiological outcomes, including scapular notching. Additionally, we were unable to account for the laterality of the procedures, which could have resulted in an over-reporting of numbers in the revision RSA complication group. The database infers that patients were on testosterone from a prescription that was filled. However, this does not take into account patients who never took the actual testosterone. Furthermore, this study did not look at the amount of testosterone that was taken, nor the exact time before surgery the testosterone was taken. Additionally, TRT can come in various types of administration and formulas. Future clinical studies should aim to collect more detailed data on TRT usage, including the dosage, duration, timing relative to surgery, and the specific form of administration, to better assess its impact on surgical outcomes. Further, another limitation is the inability to perform subgroup analyses based on baseline testosterone levels, preoperative pathologies, or comorbidity indices, as the database does not provide the necessary detailed patient-level data for such distinctions. Understanding the differences in outcomes based on this is also an important topic for future research. While the present study provides a preliminary retrospective analysis, it is crucial that more research is conducted surrounding TRT use and shoulder arthroplasty.

## 5. Conclusions

This study demonstrated that TRT use within 90 days of RSA does not increase the rates of revision, fracture, or infection. With the rise in RSA surgeries over recent decades and the increased use of testosterone for hypogonadism or gender-affirming therapy, there will be individuals on testosterone who will need this surgery. The current literature on TRT is inconclusive; it has been shown that it facilitates bone–implant integration but also increases the risk of musculotendinous complications. While RSA heavily relies on osseus implant integration, the similar revision rates in this study may be due to the idea that some patients had varying baseline testosterone levels in addition to differing long-term treatment durations, as these are both known to affect the increases in bone mineral density. Regardless, these results may assist shoulder surgeons when evaluating patients on TRT who also seek treatment for degenerative osteoarthritis, differing degrees of rotator cuff arthropathy, or severe forms of impingement. Additional research must be conducted to elucidate the temporal relationship between TRT dosing and surgical outcomes.

## Figures and Tables

**Table 1:** Comparison of patient demographics of testosterone replacement therapy (TRT) cohort and matched control cohort.

Characteristic	TRT Group ^1^	Control ^1^	*p* ^2^
Total	1906	1906	-
Age, mean	68.66	68.73	-
Sex			
Female	200 (10.4)	200 (10.5)	-
Male	1706 (89.5)	1706 (89.5)	-
Hypogonadism	1282 (67.3)	177 (9.3)	**<0.001**
Diabetes	988 (51.8)	920 (48.3)	**0.028**
Tobacco Use	927 (48.6)	904 (47.4)	0.456
Osteoarthritis	1424 (74.7)	1404 (73.7)	0.459
Chronic Kidney Disease	419 (22.0)	351 (18.4)	**0.006**
Alcohol Use	160 (8.4)	178 (9.3)	0.305
Obesity	880 (46.2)	753 (39.5)	**<0.001**
Congestive Heart Failure	206 (10.8)	195 (10.2)	0.562
Dementia	53 (2.8)	34 (1.8)	**0.039**
Decreased Libido	214 (11.2)	73 (3.8)	**<0.001**
Erectile Dysfunction	677 (35.5)	348 (18.3)	**<0.001**
Benign Prostatic Hyperplasia	645 (33.8)	492 (25.8)	**<0.001**
CCI score, mean ± SD	2.78 ± 2.35	2.78 ± 2.33	-

^1^ Data reported as n (%), unless otherwise indicated. CCI: Charlson Comorbidity Index.^2^ Statistical comparison performed using chi-squared test; bolded values are statistically significant at *p* < 0.05.

**Table 2:** Comparison of complications after reverse total shoulder arthroplasty (RSA) in testosterone replacement therapy (TRT) cohort and matched control cohort.

	TRT Group ^1^	Control ^1^	*p*
Total	1906	1906	-
Revision RSA	229 (12.0)	210 (11.0)	0.335
PJI	27 (1.4)	31 (1.6)	0.597
Implant Fracture	16 (0.84)	12 (0.63)	0.448
Periprosthetic Fracture	11 (.58)	20 (1.05)	0.105
Mechanical Loosening	46 (2.5)	43 (2.3)	0.596

^1^ Data reported as n (%), unless otherwise indicated. PJI: prosthetic joint infection.

## Data Availability

The data presented in this study are available on request from the corresponding author due to the data being part of a restricted nationwide insurance dataset.

## Supplementary Materials

The following supporting information can be downloaded at https://www.mdpi.com/article/10.3390/jcm14041341/s1, Table S1: CPT, ICD-10, and ICD-9 codes used to make populations in PearlDiver; Table S2: Exclusion criteria for TRT; Table S3: Codes used to identify patient comorbidities; Table S4: Codes for complications.

## Abbreviations

The following abbreviations are used in this manuscript:

TRT	Testosterone replacement therapy
RSA	Reverse total shoulder arthroplasty
PJI	Prosthetic joint infection
CPT	Current Procedural Terminology
ICD	International Classification of Diseases
CCI	Charlson Comorbidity Index
