# Supplementary material for: Testosterone Replacement Therapy Is Not Associated with Greater Revision Rates in Reverse Total Shoulder Arthroplasty
Source: J Clin Med. 2025 Feb 18;14(4):1341. doi: 10.3390/jcm14041341 (PMC11856249; doi:10.3390/jcm14041341)
Supplement: Supplementary file 1 [file jcm-14-01341-s001.zip › jcm-3437218-supplementary.pdf]

**Table S1**	**CPT, ICD-10, and ICD-9 Codes used to make populations in PearlDiver**
Reverse Total Shoulder Arthroplasty (rTSA)*.	ICD-9-P-8188, ICD-10-P-0RRJ00Z, ICD-10-P-0RRK00Z
Testosterone Replacement Therapy	CPT-J1070 INJECTION, TESTOSTERONE CYPIONATE, UP TO 100 MG<br>CPT-J1071 INJECTION, TESTOSTERONE CYPIONATE, 1 MG<br>CPT-J1080 INJECTION, TESTOSTERONE CYPIONATE, 1 CC, 200 MG<br>CPT-J3120 INJECTION, TESTOSTERONE ENANTHATE, UP TO 100 MG<br>CPT-J3121 INJECTION, TESTOSTERONE ENANTHATE, 1 MG<br>CPT-J3130 INJECTION, TESTOSTERONE ENANTHATE, UP TO 200 MG<br>CPT-J3140 INJECTION, TESTOSTERONE SUSPENSION, UP TO 50 MG<br>CPT-J3145 INJECTION, TESTOSTERONE UNDECANOATE, 1 MG<br>CPT-J3150 INJECTION, TESTOSTERONE PROPIONATE, UP TO 100 MG<br>CPT-J0900 INJECTION, TESTOSTERONE ENANTHATE AND ESTRADIOL<br>CPT-J1060 INJECTION, TESTOSTERONE CYPIONATE AND ESTRADIOL

	The below medications already have pre-defined cohorts within PearlDiver database:<br><br>DRUG-4-DIHYDROTESTOSTERONE, DRUG-DEPO-TESTOSTERONE, DRUG-ESTROGEN-METHYLTESTOSTERONE, DRUG-ESTROGEN_&_METHYLTESTOSTERONE, DRUG-FIRST-TESTOSTERONE, DRUG-FIRST-TESTOSTERONE_MC, DRUG-METHYLTESTOSTERONE, DRUG-METHYLTESTOSTERONE_MICRONIZED, DRUG-TESTOSTERONE, DRUG-TESTOSTERONE_CYPIONATE, DRUG-TESTOSTERONE_CYPIONATE_MICRO, DRUG-TESTOSTERONE_ENANTHATE, DRUG-TESTOSTERONE_MICRONIZED, DRUG-TESTOSTERONE_PROPIONATE

* CPT codes were unable to be used to identify patients who underwent rTSA, because the current codes used in shoulder arthroplasty (23470 and 23472) include all forms of shoulder arthroplasty

**Table S2: Exclusion Criteria for TRT**		
	Marfan Syndrome	ICD-10-D-Q8740, ICD-10-D-Q87410, ICD-10-D-Q87418, ICD-10-D-Q8742, ICD-10-D-Q8743
	Ehlers-Danlos Syndrome	ICD-10-D-Q796, ICD-10-D-Q7960, ICD-10-D-Q7961, ICD-10-D-Q7962, ICD-10-D-Q7963, ICD-10-D-Q7969
	Rheumatoid Arthritis	ICD-10-D-M069, ICD-10-D-M0579, ICD-10-D-M0609, ICD-10-D-M059, ICD-10-D-M0600, ICD-10-D-M0689, ICD-10-D-M0589, ICD-10-D-M0540, ICD-10-D-M0800, ICD-10-D-M0570, ICD-10-D-M0680, ICD-10-D-M0580, ICD-10-D-M083, ICD-10-D-M0569, ICD-10-D-M0550, ICD-10-D-M0560, ICD-10-D-M06861, ICD-10-D-M05761,

		ICD-10-D-M06862, ICD-10-D-M06061, ICD-10-D-M0820, ICD-10-D-M05762, ICD-10-D-M06062, ICD-10-D-M0809, ICD-10-D-M0559, ICD-10-D-M05712
	SLE	ICD-10-D-M329, ICD-10-D-M3210, ICD-10-D-M3219, ICD-10-D-M328, ICD-10-D-M3214, ICD-10-D-M320, ICD-10-D-M3213, ICD-10-D-M3212, ICD-10-D-M3215, ICD-10-D-M3211
	Sjogren Syndrome	ICD-10-D-M3500, ICD-10-D-M3501, ICD-10-D-M3502, ICD-10-D-M3503, ICD-10-D-M3504, ICD-10-D-M3509
	Dermatomyositis	ICD-10-D-M3310, ICD-10-D-M3313, ICD-10-D-M3312, ICD-10-D-M3300, ICD-10-D-M3319, ICD-10-D-M3311, ICD-10-D-M3302, ICD-10-D-M3309, ICD-10-D-M3301, ICD-10-D-M3303
	Polymyositis	ICD-10-D-M3320, ICD-10-D-M3321, ICD-10-D-M3322, ICD-10-D-M3329, ICD-10-D-M3390, ICD-10-D-M3391, ICD-10-D-M3392, ICD-10-D-M3393, ICD-10-D-M3399
	Mitochondrial Disease	ICD-9-D-27787, ICD-10-D-E8840, ICD-10-D-E8849, ICD-10-D-G713

Table S3. Codes used to identify patient comorbidities

	**ICD-9 and ICD-10 Codes**
**Hypogonadism**	ICD-9-D-2563x, ICD-9-D-2562, ICD-9-D-627x, ICD-9-D-V4981, ICD-9-D-2572, ICD-9-D-2571, ICD-9-D-

	2578, ICD-9-D-2579, ICD-10-D-E283x, ICD-10-D-E894, ICD-10-D-N95x, ICD-10-D-Z780, ICD-10-D-E291, ICD-10-D-E895, ICD-10-D-E298, ICD-10-D-E299
**Erectile Dysfunction**	ICD-9-D-60784, ICD-9-D-30272, ICD-10-D-N5201, ICD-10-D-N5202, ICD-10-D-N5203, ICD-10-D-N521, ICD-10-D-N522, ICD-10-D-N5231- ICD-10-D-N5232, ICD-10-D-N5233, ICD-10-D-N5234, ICD-10-D-N5235, ICD-10-D-N5236, ICD-10-D-N5237, ICD-10-D-N5239, ICD-10-D-N528, ICD-10-D-N529
**Decreased Libido**	ICD-9-D-30270, ICD-9-D-30271, ICD-9-D-30272, ICD-9-D-30274, ICD-9-D-30275, ICD-9-D-30279, ICD-9-D-79981, ICD-10-D-R6882, ICD-10-D-F520, ICD-10-D-F5221, ICD-10-D-F5232, ICD-10-D-F524, ICD-10-D-F528, ICD-10-D-F529
**BPH**	ICD-9-D-60000, ICD-9-D-60001, ICD-9-D-60010, ICD-9-D-60011, ICD-9-D-60020, ICD-9-D-60021, ICD-9-D-60090, ICD-9-D-60091, ICD-10-N401, ICD-10-N402, ICD-10-N403
**Diabetes, Tobacco Use, Osteoarthritis, Chronic Kidney Disease, Alcohol Use, Obesity, Congestive Heart Failure, Dementia**	Pre-defined groups of codes within PearlDiver
ICD: International Classification of Diseases; BPH, benign prostatic hypertrophy;	

**Table S4: Codes for complications**	
	**CPT, ICD-9, and ICD-10 Codes**
**Prosthetic Joint Infection (PJI)**	ICD-9-D-71101, ICD-9-D-71191, ICD-9-D-99666, ICD-9-D-99667, ICD-10-D-M00011, ICD-10-D-M00111, ICD-10-D-M00211, ICD-10-D-M00811, ICD-10-D-M00012, ICD-10-D-M00112, ICD-10-D-M00212, ICD-10-D-M00812

**Revision Total Shoulder Arthroplasty**	ICD-10-P-0RRJ00Z, ICD-10-P-0RRK00Z, CPT-23473, CPT-23474
**Mechanical loosening of prosthetic joint**	ICD-10-D-T84038A, ICD-10-D-T84039A
**Broken internal joint prosthesis**	ICD-10-D-T84018A, ICD-10-D-T84019A
**Periprosthetic fracture**	ICD-10-D-M9731XA, ICD-10-D-M9732XA
CPT Current Procedural Terminology; ICD: International Classification of Diseases	
